# Cesarean Section for Placenta Previa.—Report of a Case

**Published:** 1904-12

**Authors:** R. E. Skeel

**Affiliations:** Cleveland, Ohio


### SELECTIONS.

### Cesarean Section for Placenta Previa.—Report of a Case.

By R. E. SKEEL, M. D., Cleveland, Ohio.

[*Cleveland Medical Journal*, November, 1904.]

THE case which I wish to report is that of Mrs. J. V., aged 36, whose first child was still-born prematurely but who has since that time had two children, the younger seven years of age. Her labors were not especially difficult, but after the birth of the last child she noticed some dragging pain in the right side and a decided prolapse of the uterus. I first saw her two years ago, at which time there was a freely movable right kidney, marked enteroptosis, a retrodisplaced uterus which upon exertion or standing prolapsed to the level of the internal os, and an associated cystocele and rectocele. The cervix was deeply lacerated bilaterally and greatly hypertrophied and elongated. At this time curetage, Schroeder amputation of the cervix, anterior and posterior colporrhapy and ventrosuspension of the uterus were done, the posterior portion of the fundus being stitched to the abdominal wall with two fine silk sutures. Following these procedures the patient was in good health, and I saw her infrequently until December 6, 1903, when she reported that her last menstrual period was July 13, 1903. My notes at that time stated that the uterus extended nearly to the umbilicus, its superior surface seemed very thin, but that it was freely movable and with no apparent attachment to the abdominal wall. She was seen frequently but with no further examination until February 28, 1904, when the fundus was above the umbilicus, very broad and apparently depressed in the middle line superiorly. The head could not be palpated externally or bi-manually. The entire lower segment felt normal but the same thin condition of the upper segment was still apparent.

The cervix seemed normal but was markedly high up and displaced backward. On March 13, 1904, the patient had a very severe hemorrhage after she had arisen from bed and walked across the floor. I saw her soon after and found that while she was somewhat exsanguinated, the hemorrhage had been without perceptible influence upon her pulse-rate. The fetal heart which was plainly heard was normal in character and rate. Examination showed a transverse presentation, the head to the left. The cervix was undilated, almost out of reach and displaced posteriorly nearly to the promontory of the sacrum. The cervix and vagina were snugly tamponed and the patient sent to the hospital. Here the tampon was removed and an effort was made to distinguish the extent of placental attachment over the cervix, but this was ineffectual owing to the location and rigidity of the cervix. The lower uterine segment was then tamponed and labor pains awaited. That evening conditions were entirely stationary and no pains had occurred. The next morning the cervix was somewhat softer and a Champetier de Ribes bag was introduced with difficulty and partially filled. As a result the cervix was about one-third dilated but no uterine contractions took place and the cervix still maintained its original length. Upon removal of the bag it was possible to feel the placenta over the entire circumference of the cervix and attached well above the internal os on all sides.

Withdrawal of the finger was followed by such profuse bleeding that the lower uterine segment was again hastily packed.

Taking into consideration that the child was still alive, the mother in fair condition, the cervix long and only partially dilated and reached with difficulty, Cesarean section was decided upon and performed at once with the delivery of a living child. Hemorrhage during delivery of the placenta was very free but was controlled by an assistant who compressed the broad ligaments until the uterus was sutured, when it ceased spontaneously. The mother made an uninterrupted recovery and the child is at present alive and thriving.

The condition of the uterine suspensory ligament was noted with considerable interest. It extended from the abdominal wall just above the pubes to the posterior surface of the fundus; was more than six inches in length and a finger's-breadth wide and was entirely wrapped in omentum. The only anomaly for which it seemed to be responsible was the approximation of the cervix to the sacrum, while the apparent thinning and depression of the fundus were due to the malpresentation of the fetus and absence of the placenta from its usual location.

